# *Litsea japonica* extract inhibits neuronal apoptosis and the accumulation of advanced glycation end products in the diabetic mouse retina

**DOI:** 10.3892/mmr.2015.3543

**Published:** 2015-03-24

**Authors:** JUNGHYUN KIM, CHAN-SIK KIM, YUN MI LEE, EUNJIN SOHN, KYUHYUNG JO, JIN SOOK KIM

**Affiliations:** Korean Medicine Based Herbal Drug Development Group, Herbal Medicine Research Division, Korea Institute of Oriental Medicine, Daejeon 305–811, Republic of Korea

**Keywords:** advanced glycation end products, diabetic retinopathy, *Litsea japonica*, retinal ganglion cells

## Abstract

The retinal accumulation of advanced glycation end products (AGEs) is a condition, which is found in diabetic retinopathy. The purpose of the present study was to investigate the protective effect of *Litsea japonica* extract (LJE) and to elucidate its underlying protective mechanism in model diabetic db/db mice. Male, 7 -week-old db/db mice were treated with LJE (100 or 250 mg/kg body weight) once a day orally for 12 weeks. The expression levels of AGEs and their receptor (RAGE) were subsequently assessed by immunohistochemistry. An electrophoretic mobility shift assay and southwestern histochemistry were used to detect activated nuclear factor κB (NF-κB). The immunohistochemical analysis demonstrated that LJE significantly reduced the expression levels of the AGEs and RAGE in the neural retinas of the db/db mice. LJE markedly inhibited the apop-tosis of retinal ganglion cells. In addition, LJE suppressed the activation of NF-κB. These results suggested that LJE may be beneficial for the treatment of diabetes-induced retinal neurodegeneration, and the ability of LJE to attenuate retinal ganglion cell loss may be mediated by inhibition of the accumulation of AGEs.

## Introduction

Retinal neuronal cells undergo functional alterations and cell death under diabetic conditions (1–3). Diabetic retinopathy is associated with the loss of retinal ganglion cells (RGCs), and RGC death causes permanent impairment of visual function (2,3). Previous studies have suggested that neurodegeneration undergoes two phases: Direct damage to the RGCs and secondary damage to the RGCs by the responses of non-neuronal cells. This secondary damage is considered to be the major cause of RGC loss, which occurs in diabetic retinopathy (4).

Advanced glycation end products (AGEs) are the late products of non-enzymatic glycation. The levels of these products are significantly higher in patients with diabetes (5). AGEs are important in the mechanism of diabetic retinopathy (6,7) and are accumulated in high levels in the neural retina of diabetic rats (8). AGEs can induce apoptotic cell death in retinal neuronal cells (9, and AGE-induced-apoptosis is mediated by increasing oxidative stress or via the induction of pro-apoptotic cytokines by interaction between the AGEs and their receptor (RAGE) (10–12). RAGE can recognize multiple ligands, including amyloid-β and AGEs. The over-expression of RAGE can activate the membrane-transporting system of AGE, resulting in accumulation of AGEs in the parenchyma (13).

Aminoguanidine (AG) is a selective inhibitor of AGEs, and has been found to prevent the development of diabetic retinopathy in experimental animals (14–16). In a multicenter trial, AG slowed the progression of diabetic retinopathy (17). Some medicinal plants also have an inhibitory effect on the formation of AGEs (18). *Litsea japonica* (Thunb) Jussieu is a valuable species of native Korean plant (19). This herb has been utilized as a vegetable food in Korea, however, the pharmacological activities of *L. japonica* have not yet been investigated. Chemical constituents of this plant include several types of essential oils, fatty acids, lactones, alkaloids and terpenoids (19,20). In our previous study, *L. japonica* extract (LJE) exhibited 2.9-fold higher inhibitory activity against AGE formation compared with aminoguanidine, and prevented the development of diabetic nephropathy in diabetic mice (21). Therefore, the present study examined the preventive effect of ethanol extract of *L. japonica* on diabetes-induced retinal neuronal apoptosis in db/db mouse, an animal model of type II diabetes. The present study also aimed to investigate the possible mechanism underlying the effect of *L. japonica* extract on the formation of AGE and expression of RAGE associated with the loss of retinal ganglion cells in retinal tissue.

## Materials and methods

### Preparation of LJE

The aerial parts of *L. japonica* were collected from Jeju (Republic of Korea) and identified by botanist Professor J. H. Kim (Department of Life Science, Gacheon University, Korea). A voucher specimen (no. Diab-2008–61) of the sample were deposited in the Herbarium of the Herbal Medicine Research Division, Korea Institute of Oriental Medicine (Daejeon, Korea). The dried and ground plant material (3 kg) was extracted using EtOH (3X, 20 litres; Duksan Pure Chemicals Co., Ltd., Ansan, Korea) by maceration at room temperature for 3 days. The extracts were combined and concentrated *in vacuo* at 40°C to produce an EtOH extract (390 g).

### HPLC analysis

LJE (10 mg) was dissolved in MeOH (10 ml; Duksan Pure Chemicals Co., Ltd.) and the solution was filtered through a 0.2 μm syringe filter (Millipore, Bedford, MA, USA) prior to injection. Each analysis was repeated three times and calibration curves were fitted by linear regression (LC solution version 1.25 software, LC-10AD series HPLC system; Shimadzu, Kyoto, Japan).

### Animals and experimental design

Male C57BL/KsJ db/db mice (db/db) and their age-matched non-diabetic littermates (db/+) were purchased from Japan SLC, Inc. (Shizuoka, Japan). Mice were housed four per cage in a 12-h light/12-h dark cycle at a temperature of 23±1°C and provided with food and water *ad libitum*. At 8-weeks of age, the db/db mice were randomly assigned into four groups (n=10). In one group, the LJE was dissolved in vehicle (0.5% w/v carboxyl methylcellulose solution; Sigma-Aldrich, St. Louis, MO, USA) at a concentration of 5 mg/ml. Two groups of the db/db mice received daily gastric gavage of LJE at 100 or 250 mg/kg, respectively, and the fourth group was administered with the same quantity of vehicle gavage for 12 weeks. The non-diabetic littermates received the same vehicle treatment. The blood glucose level was monitored consecutively, and glycated hemoglobin (HbA1c) was determined using a commercial kit (Unimate HbA1c; Roche Diagnostics, Mannheim, Germany). At necropsy, the eye from each mouse was enucleated under deep anesthesia, following intraperitoneal injection of pentobarbital sodium (30 mg/kg body weight; Hanlim Pharmaceuticals Inc., Seoul, Korea), fixed in 10% neutralized formalin for 24 h and embedded in paraffin (Thermo Fisher Scientific, Pittsburgh, PA, USA). Animals were then sacrificed with an overdose of pentobarbital sodium (200 mg/kg body weight; Hanlim Pharmaceuticals Inc.). All procedures involving animals were performed in accordance with the Association of Research in Vision and Ophthalmology statement for the Use of Animals in Ophthalmic and Vision Research, and were approved by the Korea Institute of Oriental Medicine Institutional Animal Care and Use Committee (Daejeon, Korea).

### Apoptosis assay

To evaluate apoptosis in retinal neuronal cells, a terminal deoxynucleotidyl transferase dUTP nick end labeling (TUNEL) assay was performed using a DeadEnd apoptosis detection system (Promega Corporation, Madison, WI, USA), according to the manufacturer’s instructions. Biotinylated dUTPs were recognized by fluorescein-conjugated streptavidin (Santa Cruz Biotechnology, Inc., Santa Cruz, CA, USA) at 1:500 in PBS for 30 min at room temperature. Images were captured using an Olympus BX51 microscope and DP71 digital camera (Olympus, Tokyo, Japan). For quantitative analysis, the TUNEL-positive nuclei in the ganglion cell layer were counted on each side of the optic nerve. The counts from the two sides were averaged and reported per unit length (1 mm).

### Immunohistochemical staining

Immunohistochemistry was performed, as previously described (22). The following antibodies were used: Monoclonal mouse anti-AGEs (1:200, cat. no. KAL-KH001; Cosmo Bio Co, Ltd., Tokyo, Japan) and polyclonal rabbit anti-mouse RAGE (1:200; cat. no. SC-5563; Santa Cruz Biotechnology, Inc.). For the detection of AGEs and RAGE, the sections were incubated with a labeled streptavidin-biotin kit (DAKO, Carpinteria, CA, USA) and were visualized by 3,3′-diaminobenzidine tetrahydrochloride. Images were captured using an Olympus BX51 microscope and DP71 digital camera (Olympus). For morphometric analysis, the positive signal intensity per unit area (0.32mm^2^) in a total of 10 randomly selected fields were determined using Image J software (version 1.48; National Institutes of Health, Bethesda, MD, USA).

### Measuring nuclear factor-κB (NF-κB) activity

For the electrophoretic mobility shift assay (EMSA), nuclear extracts were prepared with a kit according to the manufacturer’s instructions (NE-PER™ nuclear and cytoplasmic extraction reagents; Pierce Biotechnology, Inc., Rockford, IL, USA). The EMSA assay was performed by incubating 10 μg nuclear protein extract with IRDye 700-labeled NF-κB oligonucleotide (LI-COR Biosciences, Lincoln, NE, USA) or an unlabeled NF-κB probe (Promega Corporation) for cold competition. The EMSA gels were analyzed and images were captured and quantified using a LI-COR Odyssey infrared laser imaging system (LI-COR Biosciences).

### Southwestern histochemistry for the detection of activated NF-κB

To localize the activity of NF-κB in the retina, *in situ* southwestern histochemistry was performed, as described by Hernandez-Presa *et al* (23). The intensity of the cells positive to NF-κB activation in the ganglion cell layer were then counted using computer assisted Image J software (version 1.48; National Institutes of Health). Negative control groups included: The absence of a probe, a mutant digoxigenin-labeled NF-κB probe, and competition assays with a 200-fold excess of unlabeled NF-κB, followed by incubation with the labeled probe.

### Statistical analysis

Statistical analyses of the results were performed using Student’s t-test an one-way analysis of variance, followed by Tukey’s multiple comparison test, using GraphPad Prism 4.0 software (GraphPad Software, Inc., La Jolla, CA, USA). Data are expressed as the mean ± standard error of the mean. P<0.01 was considered to indicate a statistically significant difference.

## Results

### HPLC analysis of LJE

To determine the quality of the LJE, HPLC analysis was performed. The major compounds of LJE were epicatechin, quercitrin and afzelin, and the contents of these compounds were 11.53±0.023, 3.96±0.003 and 7.73±0.011 mg/g, respectively.

### Levels of blood glucose and HbA1c

At 20 weeks of age, all the db/db mice had developed hyperglycemia compared with the non-diabetic mice. Treatment with LJE caused a marginal decrease in blood glucose levels, and no significant reduction in the levels of HbA1c was observed in the db/db mice (Table I).

### LJE inhibits the formation and accumulation of AGEs and reduces the expression of RAGE

**Figure 1:**
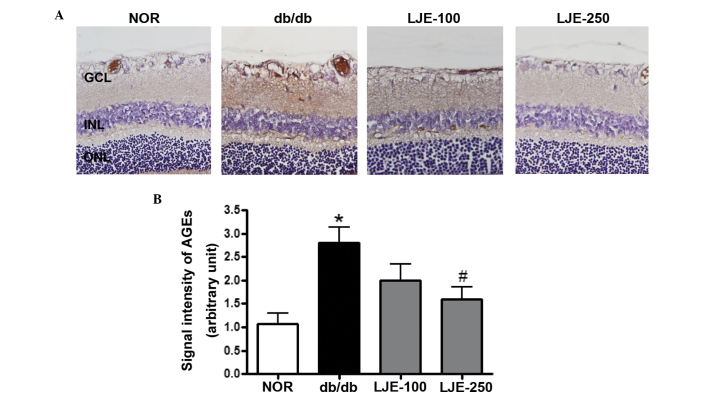
Accumulation of AGEs. (A) Representative immunostaining of AGEs in the retina (magnification, ×200). db/db, diabetic db/db mice; LJE-100, db/db mice treated with LJE (100 mg/kg); LJE-250, db/db mice treated with LJE (250 mg/kg). (B) Quantitative analysis of AGE signal intensity. Values are expressed as the mean ± standard error of the mean, n=8. ^*^P<0.01, vs. NOR mice, ^#^P<0.01, vs. vehicle-treated db/db mice. GCL, ganglion cell layer; INL, inner nuclear layer; ONL, outer nuclear layer; AGEs. advanced glycation end products; NOR. normal; LJE, *Litsea japonica* extract.

**Figure 2:**
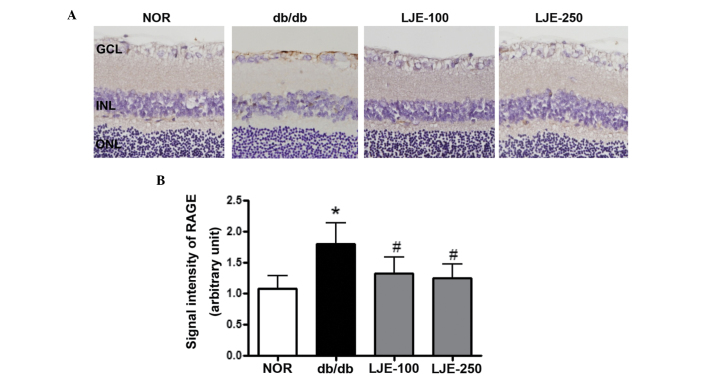
Expression of RAGE. (A) Representative immunostaining of RAGE in the retina (magnification, ×200). db/db, diabetic db/db mice; LJE-100, db/db mice treated with LJE (100 mg/kg); LJE-250, db/db mice treated with LJE (250 mg/kg). (B) Quantitative analysis of RAGE signal intensity. Values are expressed as the mean ± standard error of the mean, n=8. ^*^P<0.01, vs. NOR mice; ^#^P<0.01, vs. vehicle-treated db/db mice. GCL, ganglion cell layer; INL, inner nuclear layer; ONL, outer nuclear layer; RAGE, receptor for advanced glycation end products; NOR. normal; LJE, *Litsea japonica* extract.

LJE was assessed for its ability to inhibit the formation and accumulation of AGEs in the retina by performing immunohistochemical staining for AGEs at the end of the investigation. The immunoreactivity of AGE was only observed in the large and small retinal vessels of the normal mice, whereas AGE-positive signals were located in the retinal vessels and the inner neural retina in the vehicle-treated db/db mice, indicating that serum AGEs had accumulated in the retinal tissues. However, treatment with LJE reduced the AGE deposited in these regions (Fig. 1). The inhibitory effect of LJE on the expression of RAGE was also examined. Immunohistochemical staining for RAGE revealed that the extent of retinal RAGE immunolabeling was higher in the vehicle-treated db/db mice compared with the normal mice (Fig. 2). The quantitative analysis demonstrated that the expression of RAGE increased 1.8-fold in the vehicle-treated db/db mice compared with the normal mice, and these changes were reduced following treatment with LJE.

### Apoptosis in retinal neuronal cells

**Figure 3:**
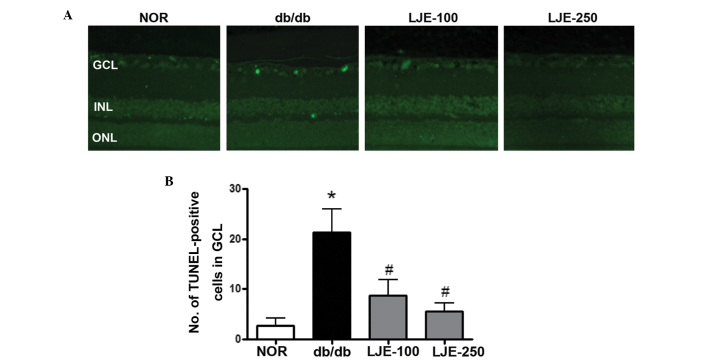
Apoptosis of retinal ganglion cells. (A) Retinal sections were stained with TUNEL (green). Apoptotic ganglion cells were observed in the vehicle-treated db/db mice (magnification, ×200). db/db, diabetic db/db mice; LJE-100, db/db mice treated with LJE (100 mg/kg); LJE-250, db/db mice treated with LJE (250 mg/kg). (B) Quantitative analysis of TUNEL-positive cells in GCL. Values are expressed as the mean ± standard error of the mean, n=8. ^*^P<0.01, vs. NOR mice, ^#^P<0.01, vs. vehicle-treated db/db mice. TUNEL, terminal deoxynucleotidyl transferase dUTP nick end labeling; GCL, ganglion cell layer; INL, inner nuclear layer; ONL, outer nuclear layer; NOR, normal; LJE, *Litsea japonica* extract.

To characterize the death of neurons in the ganglion cell layer (GCL) of the vehicle-treated db/db mice, TUNEL staining was performed. A significant increase in TUNEL-positive cells were observed in the GCLs of the vehicle-treated db/db mice compared with the normal mice (Fig. 3). The presence of TUNEL-positive cells was not limited to the GCL. The inner nuclear layer demonstrated occasional positive cells, as did the outer nuclear layer of the photoreceptor cell nuclei. However, treatment of the db/db mice with LJE prevented an increase in positive cells, similar to that observed in the normal mice.

### LJE inhibits the activation of NF-κB in the retina

**Figure 4:**
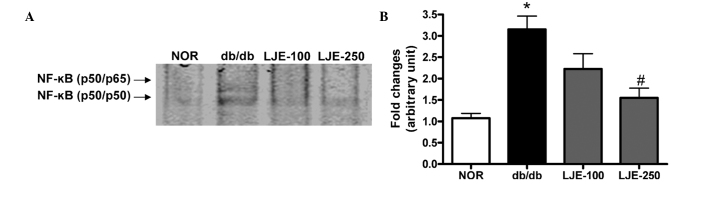
Activation of NF-κB. (A) The NF-κB DNA-binding activity was measured by EMSA. db/db, diabetic db/db mice; LJE-100, db/db mice treated with LJE (100 mg/kg); LJE-250, db/db mice treated with LJE (250 mg/kg) (B) Data are expressed as the mean ± standard error of the mean, n=8. ^*^P<0.01, vs. NOR mice, ^#^P<0.01, vs. vehicle-treated db/db mice. NF-κB, nuclear factor-κB.. NOR, normal; LJE, *Litsea japonica* extract.

**Figure 5:**
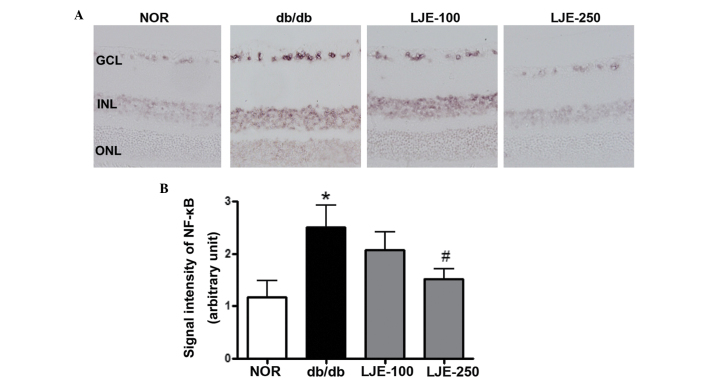
SWH for *in situ* detection of active NF-κB. (A) Positive signals for activated NF-κB were predominantly detected in the nuclei of the retinal ganglion cells and inner nuclear cells in the vehicle-treated db/db mice. (magnification, ×200). db/db, diabetic db/db mice; LJE-100, db/db mice treated with LJE (100 mg/kg); LJE-250, db/db mice treated with LJE (250 mg/kg). (B) Quantitative analysis of SWH-positive signal intensity. Data are expressed as the mean ± standard error of the mean, n=8. ^*^P<0.01, vs. NOR mice, ^#^P<0.01, vs. vehicle-treated db/db mice. SWH. southwestern histochemistry; NF-κB, nuclear factor-κB; GCL, ganglion cell layer; INL, inner nuclear layer; ONL, outer nuclear layer; NOR. normal; LJE, *Litsea japonica* extract.

NF-κB is a common downstream signal of AGEs (24). Since inhibition of NF-κB activity is considered one of the mechanisms promoting apoptosis (25,26), the present study examined whether LJE inhibited the activation of NF-κB. EMSA analysis of the nuclear protein revealed consistently increased DNA binding activity of NF-κB in the vehicle-treated db/db mice compared with the normal mice, with a 3.1-fold increase (P<0.01; Fig. 4). LJE significantly inhibited the DNA binding activity of NF-κB. In addition, southwestern histochemistry was performed to determine the activity of NF-κB the retinal tissue. This technique allows the localization of activated nuclear factor in the cellular nucleus and, using this novel method, marked NF-κB activity was found predominantly in the nuclei in the GCL and in the inner nuclear layer in the vehicle-treated db/db mice (Fig. 5A). Morphometric analysis revealed that the expression of activated NF-κB in the vehicle-treated db/db mice was significantly increased compared with the normal mice, whereas LJE significantly inhibited the activated NF-κB (Fig. 5B).

## Discussion

In streptozotocin-induced diabetic rats and postmortem human retinas, TUNEL assays have revealed that diabetes increases apoptosis in neurons, particularly in the inner retina, where retinal ganglion cells are located (27,28). The rate of neural apoptosis remains constant throughout the duration of diabetes (3). As neurons are unable to proliferate, the apoptosis of these cells leads to chronic neurodegeneration. In the present study, the preventive effect of LJE on diabetes-induced injury of retinal ganglion cells was evaluated. LJE, a herbal AGE inhibitor, reduced diabetes-induced apoptosis of the retinal ganglion cells. In addition, LJE prevented AGE accumulation in the neural retina and decreased the expression of RAGE in the retinal tissues. LJE marginally decreased the levels of blood glucose, but caused no reduction in the levels of HbA1c in the db/db mice. Therefore, LJE had anti-apoptotic effects in the diabetic neural retinas without a substantial reduction in blood glucose. These results suggested that, even in hyperglycemia, diabetes-induced retinal neurodegeneration was attenuated by LJE.

The potential mechanisms of diabetic retinopathy are numerous. The accumulation of AGEs during the Maillard reaction is associated with the risk of diabetic retinopathy and levels of serum AGEs correlate with the degree of diabetic retinopathy (29,30). In addition, retinal ganglion cells are the most vulnerable cell population in the retina (31). AGE-RAGE interaction elicits the induction of apoptosis in various types of cell (12,32,33) and the inhibition of AGE formation improves diabetic retinopathy (32–34). In the present study, LJE exhibited the properties of an AGE inhibitor in retinal tissue, and treatment with LJE resulted in a decline in the cellular damage mediated by AGEs. In the present study, three flavonoids (epicatechin, quercitrin and afzelin) were identified in LJE. These flavonoids exhibit significant inhibitory activities on the formation of AGEs, with previously reported half maximal inhibitory concentrations of 144, 77.8 and 58.9 μM, respectively (35–37). Therefore, the ability of LJE to protect against retinal neurodegeneration may be due to the effect of this compound.

Notably, NF-κB has been previously implicated in the development of diabetic retinopathy (25). AGEs interact with RAGE, inducing the subsequent activation of NF-κB and NF-κB-controlled pro-apoptotic molecules (38). Apoptosis of retinal pericytes and the retinal neuronal cells is also associated with NF-κB (25,26). Although the activation of NF-κB in the retina may be involved in retinal cell death or survival (39,40), the activation of NF-κB due to hyperglycemia induces the accelerated loss of retinal pericyts (25) and retinal capillary cell death (26). In the present study, southwestern histochemistry demonstrated that NF-κB was markedly activated in the ganglion cell layer of the db/db mice. This result suggested that the activation of NF-κB activation was responsible for the loss of ganglion cells. Treatment with LJE almost completely inhibited this activation of NF-κB.

In conclusion, LJE suppressed the accumulation of AGEs in neural retinas. In addition, the expression of RAGE, which is important in the pro-apoptotic signaling pathway, was restored. Although the pathogenesis of diabetic nephropathy is multifactorial, AGE/RAGE signaling is a common pathway for the progression of diabetic retinopathy. Therefore, the neural protective effect of LJE may be, at least partly, attributed to its effect on AGEs, and LJE may be a beneficial agent in protecting against diabetes-induced retinal neurodegeneration.

## Figures and Tables

**Table I:** Levels of blood glucose and HbA1c in different groups of mice.

Factor	Normal	db/db	LJE-100	LJE-250
Blood glucose (mmol/l)	6.7±2.0	43.0±0.8^a^	38.6±9.8	32.5±12.0
HbA1c (%)	3.6±0.1	7.3±0.8	7.9±0.9	7.3±1.3

db/db, diabetic *db/db* mice; LJE-100, db/db mice treated with LJE (100 mg/kg); LJE-250, db/db mice treated with LJE (250 mg/kg). All data are expressed as the mean ± standard error of the mean.

a P<0.01, vs. the normal group; ^b^P<0.01, vs. the db/db group. LJE, *Litsea japonica* extract; HbA1c, glycated hemoglobin.
